# Relationship of Health Services to Medical Expenses for the National Health Insurance and Certification Rate for Long-term Care Insurance Services in Municipalities

**DOI:** 10.2188/jea.12.136

**Published:** 2007-11-30

**Authors:** Atsushi Hioki

**Affiliations:** Gifu Prefectural Gifu Region Public Health Center, Gifu, Japan.

**Keywords:** health service, health examination, cancer screening, medical expense, long-term care service

## Abstract

In many municipalities, implementation rates of health services mandated by the Health and Medical Service Law for the Elderly have not reached the national goal that was set at the start. This study aimed to evaluate the effects of health services using medical expenses for the National Health Insurance (NHI) and certification rate for long-term care insurance services in 99 municipalities in Gifu Prefecture as indices. Both indices were standardized by the age composition of the population. Among the health services, visit rates for health examinations or implementation rates for health education or health counseling correlated negatively with medical expenses for each insured person. The visit rate for gastric cancer screening correlated negatively with medical expenses for malignant neoplasms of the stomach. Implementation rates of health education or health counseling, or ratios of public health nurses correlated positively with certification rates for long-term support need and care need grade 1, and negatively with those for long-term care need grades 2, 3, and 4. The author concluded that medical expenses are reduced by the implementation of available health services, that early detection and prevention of aggravation of disease is essential for those who need long-term care services, and that health services must be reinforced with primary prevention.

## INTRODUCTION

In Japan, the Health and Medical Service Law for the Elderly was enforced to cope with a shift from infectious to chronic diseases in 1983^1^^)^. Municipal governments issue health handbooks and provide health education, health counseling, health examinations, functional training, and home-visit guidance to residents aged 40 years and older based on health services required by law. Recently, the implementation rates of these health services have remained at a lower level than the national goal in many municipalities in Gifu Prefecture, although these services were expanded vigorously by municipal governments at the beginning. A review of available health services will be necessary to determine whether reinforcement is necessary.

In 2000, the “National Health Promotion Movement in the 21st Century (Healthy Japan 21)” was initiated to promote comprehensive health through a decrease in premature death and an extension of the period during which people can live without suffering dementia or being bedridden. The above-mentioned health services were instituted as part of the plan to realize Healthy Japan 21^1^^)^. Simultaneously, following a six-month trial, the municipalities started a long-term care insurance system to provide social support for long-term care for the elderly and to enable them to maintain their dignity by keeping up their self-constituted lives.

The aim of health services in the municipalities is social support for individual health initiatives to enhance people’s capacities for living^2^^)^. In order to evaluate the effects of these services, the fullness of individual life must be used as an index. This is difficult to measure, however, because of its subjectivity and, thus, it cannot be used as a factor to increase a budget for health services in the municipalities. The usual measurement of the effects of health services is the relationship to medical expense^3^^-^^8^^)^. In this study, the author analyzed the relationship of implementation rates of health services to medical expenses per person for National Health Insurance (NHI) and certification rate for long-term care insurance services for each resident.

## MATERIALS AND METHODS

Gifu Prefecture is located in the center of Japan and comprises 99 municipalities: 14 cities, 55 towns and 30 villages. The prefecture had a population of 2.1 million in 1995^9^^)^ within an area of 10,595.75 km^2^. The percentage of the population aged 65 years and older was 15.3%.

### Data source

The data on health services used in this study were visit rates for health examinations^10^^)^, implementation rates of health education, health counseling, and home-visit guidance^11^^)^ in fiscal years 1995-9, and ratios of public health nurses at work per population of each municipality from 1996-2000. The ratios of hospital beds per population of each secondary emergency medical service area from 1996-2000, and ratios of physicians and dentists at work per population of each municipality and each secondary emergency medical service area in 1996 and 1998^11^^)^ were used for data on medical supplies. Population density from 1996-2000 and per capita income by municipality from 1994-8^12^^)^ were used as socioeconomic data. Medical expenses for NHI in the municipalities in May 1996-2000^13^^)^, and the number of persons certified for long-term care insurance services on May 31, 2000, in the municipalities^14^^)^ were used as indices of the effects of health services.

Health examinations include basic health examinations mandated by law, and gastric, uterine, lung, breast, and colorectal cancer screenings. These health examinations are provided for residents aged 40 years and older who are not offered such examinations at their workplaces or elsewhere. Of all residents in Gifu Prefecture aged 40 years and older, 33.4% were the subjects for basic health examinations from 1995-9. The implementation rates of health education, health counseling, and home-visit guidance were calculated as figures per subject for basic health examinations.

Medical expense per insured person was standardized for 5-year age group by the indirect method using the total population of Gifu Prefecture as the standard. The ratio of the subjects of the NHI from 1996-2000 was 41.2% of all residents in Gifu Prefecture. Included in this study were malignant neoplasms of the stomach, uterus, trachea/bronchus/lung, breast, and large intestine; diabetes mellitus; circulatory, hypertensive, heart, and cerebrovascular diseases; medical outpatients, medical inpatients, medical total; and dental total.

In Japan, the conditions requiring long-term care insurance services are classified into long-term support need and care need grades 1-5. The certification rate per resident aged 65 years and older was also standardized for age groups 65-74 and 75+.

### Statistical Analysis

The coefficient of variation (CV) for each index was calculated to examine the annual variation for 5 years in each municipality. Data correlations were calculated for all municipalities. Stepwise regression analyses (F_in_ = 4.000, F_out_ = 3.996) were then conducted using the medical expense or the certification rate for long-term care services as the dependent variables, and the indices for health services, medical supplies and socioeconomic factors that correlated with each dependent variable as the independent variables.

## RESULTS

The ratios of municipalities that reached the national goals of visit rates for health examinations (50% for basic health examinations and 30% for cancer screenings) were 63.6%, 37.4%, 12.1%, 46.5%, 24.2%, and 37.4% for basic health examinations, gastric, uterine, lung, breast, and colorectal cancer screenings, respectively. Fifteen municipalities did not conduct lung cancer screenings. Table 1 shows the characteristics of indices for health services and medical expenses. CVs for the visit rates for health examinations remain at stationary levels in many municipalities. Only the CV for medical expenses for malignant neoplasms exceeded 50.

**Table 1.** Characteristics of indices for health services and medical expenses.

Indices	Figures for Gifu Prefecture	Mean and 95% CI of coefficients of variation for 5 years in the municipalities
Visit rate, %		
Basic medical examinations	40.2	10.7 (9.1-12.3)
Gastric cancer screening	15.1	18.2 (15.7-20.7)
Uterine cancer screening	13.8	17.9 (15.5-20.3)
Lung cancer screening	25.8	33.3 (24.4-42.3)
Breast cancer screening	13.5	19.6 (16.6-22.7)
Colorectal cancer screening	14.3	20.8 (17.9-23.7)
Implementation rate, times per person		
Health education	0.49	34.3 (30.4-38.2)
Health counseling	0.36	28.6 (25.2-32.0)
Home-visit guidance	0.034	44.6 (39.4-49.9)
Medical expense*, yen per person		
Medical total	22,060	10.2 (9.1-11.3)
Medical outpatient	11,228	6.0 (5.5-6.6)
Medical inpatient	10,831	18.1 (16.0-20.1)
Malignant neoplasms	2,327	36.8 (32.5-41.2)
Stomach	475	75.3 (67.0-83.6)
Uterus	80	126.7 (114.5-138.8)
Trachea, bronchus and lung	275	95.4 (84.6-106.1)
Breast	128	91.8 (82.4-101.2)
Large intestine	417	76.0 (67.8-84.2)
Diabetes mellitus	759	31.1 (26.9-35.2)
Circulatory diseases	7,549	17.0 (15.0-18.9)
Heart diseases	2,545	37.2 (32.8-41.5)
Hypertensive diseases	1,993	12.9 (11.6-14.1)
Cerebrovascular diseases	2,768	30.5 (26.6-34.4)
Dental total	1,755	13.0 (11.3-14.6)

* Monthly medical expense per insured person in May 1996-2000.

Visit rates for 6 kinds of health examinations, and implementation rates of health education, health counseling, and home-visit guidance correlated positively with each other (Table 2). The ratio of public health nurses correlated positively with visit rates and especially with implementation rates. Population density and per capita income correlated negatively with visit rates for health examinations and the ratio of hospital beds in secondary emergency medical service area correlated positively (Table 3). The ratio of public health nurses correlated negatively with population density and the ratio of physicians and dentists correlated positively.

**Table 2.** Correlation between indices of health services in municipalities.

	VR* for basic health examinations	VR for gastric cancer screening	VR for uterine cancer screening	VR for lung cancer screening	VR for breast cancer screening	VR for colorectal cancer screening	IR* of health education	IR of health counseling	IR of home-visit guidance
VR for gastric cancer screening	0.71^§^								
VR for uterine cancer screening	0.58^§^	0.76^§^							
VR for lung cancer screening	0.69^§^	0.68^§^	0.53^§^						
VR for breast cancer screening	0.55^§^	0.77^§^	0.88^§^	0.48^§^					
VR for colorectal cancer screening	0.68^§^	0.79^§^	0.71^§^	0.58^§^	0.73^§^				
IR of health education	0.20	0.28^†^	0.26^†^	0.10	0.37^‡^	0.34^‡^			
IR of health counseling	0.27^†^	0.29^†^	0.27^†^	0.11	0.35^‡^	0.31^†^	0.77^§^		
IR of home-visit guidance	0.31^†^	0.35^‡^	0.23	0.27^†^	0.36^‡^	0.32^†^	0.75^§^	0.75^§^	
Ratio of public health nurses	0.44^§^	0.52^§^	0.33^‡^	0.40^§^	0.43^§^	0.49^§^	0.64^§^	0.69^§^	0.79^§^

*VR, visit rate; IR, implementation rate.

^†^p < 0.01, ^‡^p < 0.001, ^§^p < 0.0001.

**Table 3.** Correlation between indices of health services, medical supplies and socioeconomic factors in municipalities.

	VR* for basic health examinations	VR for gastric cancer screening	VR for uterine cancer screening	IR* of health education	Ratio of public health nurses	Ratio of hospital beds in area*	Ratio of physicians in area	Ratio of dentists in area	Population density
VR for gastric cancer screening	0.71^§^								
VR for uterine cancer screening	0.58^§^	0.76^§^							
IR of health education	0.20	0.28^†^	0.26^†^						
Ratio of public health nurses	0.44^§^	0.52^§^	0.33^‡^	0.64^§^					
Ratio of hospital beds in area	0.41^§^	0.35^‡^	0.42^§^	-0.01	-0.01				
Ratio of physicians in area	0.13	0.01	0.15	-0.10	-0.14	0.76^§^			
Ratio of dentists in area	-0.35^‡^	-0.44^§^	-0.32^†^	-0.13	-0.23	-0.01	0.62^§^		
Population density	-0.47^§^	-0.44^§^	-0.25	-0.29^†^	-0.43^§^	0.04	0.43^§^	0.62^§^	
Per capita income	-0.36^‡^	-0.26^†^	-0.08	0.03	-0.18	0.03	0.25	0.34^‡^	0.52^§^

*VR, visit rate; IR, implementation rate; area, secondary emergency medical service area.

^†^p < 0.01, ^‡^p < 0.001, ^§^p < 0.0001.

Regarding medical expenses, visit rates for health examinations, especially those for lung cancer screening, and implementation rates of health education correlated negatively with medical expenses for outpatients and the ratio of physicians correlated positively (Table 4). Implementation rates of health counseling correlated negatively with medical expenses for inpatients and the ratio of physicians correlated positively. The ratio of public health nurses correlated negatively with medical expenses for diabetes mellitus and the ratio of physicians correlated positively. The ratio of public health nurses and physicians correlated positively with medical expenses for hypertensive diseases and per capita income correlated negatively. The implementation rate of health education correlated negatively with medical expenses for cerebrovascular diseases and popular density correlated positively. The ratio of public health nurses correlated negatively with expenses for dental diseases and per capita income and the ratio of dentists correlated positively. No implementation rate of health services correlated with medical expenses for either circulatory or heart disease (data not shown).

**Table 4.** Relationship of health services, medical supplies and socioeconomic factors to medical expenses.

	Medical outpatient	Medical inpatient	Diabetes mellitus	Hypertensive diseases	Cerebrovascular diseases	Dental total
Visit rate for health examinations	-0.53^‡^	0.18	-0.19	0.05	-0.20*	-0.49^‡^
	-0.53^‡^	NU	NU	NU	NS	NS

Implementation rate of health education	-0.27^†^	-0.14	-0.26^†^	0.08	-0.32^†^	-0.22*
	-0.17*	NU	NS	NU	-0.23*	NS

Implementation rate of health counseling	-0.12	-0.22*	-0.28^†^	0.14	-0.27^†^	-0.19
	NU	-0.20*	NS	NU	NS	NU

Implementation rate of home-visit guidance	-0.20*	-0.08	-0.35^‡^	0.10	-0.26*	-0.28^†^
	NS	NU	NS	NU	NS	NS

Ratio of public health nurses	-0.18	-0.03	-0.38^‡^	0.23*	-0.32^†^	-0.48^‡^
	NU	NU	-0.35^‡^	0.22*	NS	-0.34^‡^

Population density	0.50^‡^	0.09	0.30^†^	-0.12	0.37^‡^	0.54^‡^
	NS	NU	NS	NU	0.30^†^	NS

Per capita income	0.33^‡^	0.04	0.25*	-0.27^†^	0.24*	0.47^‡^
	NS	NU	NS	-0.31^†^	NS	0.27^‡^

Ratio of hospital beds	-0.09	0.26^†^	-0.00	-0.15	0.13	-0.31^†^
	NU	NS	NU	NU	NU	NS

Ratio of physicians or dentists	0.33^‡^	0.30^†^	0.25*	0.30^†^	0.24*	0.58^‡^
	0.35^‡^	0.29^†^	0.20*	0.38^‡^	NS	0.41^‡^

Multiple correlation coefficient						
	0.66^‡^	0.36^†^	0.43^‡^	0.50^‡^	0.43^‡^	0.73^‡^

Upper shows correlation coefficient; lower, standardized regression coefficient. NU, not used for stepwise regression analysis; NS, not selected by stepwise regression analysis.

*p < 0.05, ^†^p < 0.01, ^‡^p < 0.001.

The visit rate for lung cancer screening correlated negatively with medical expenses for malignant neoplasms and the ratio of physicians correlated positively (Table 5). The visit rate for gastric cancer screening correlated negatively with medical expenses for malignant neoplasms of the stomach and population density correlated positively. The ratio of public health nurses correlated negatively with medical expenses for malignant neoplasms of the uterus and the ratio of physicians correlated positively. The ratio of public health nurses correlated negatively with medical expenses for malignant neoplasms of the lung. Neither medical expenses for malignant neoplasms of the breast nor those of the large intestine showed a correlation with the indices used in this study.

**Table 5.** Relationship of health services, medical supplies and socioeconomic factors to medical expenses for malignant neoplasms.

	Malignant neoplasms	Stomach	Uterus	Trachea, bronchus and lung	Breast	Large intestine
Visit rate for the corresponding cancer screenings	-0.37^‡^	-0.37^‡^	-0.01	-0.20*	-0.02	-0.10
	-0.39^‡^	-0.27^†^	NU	NS	NU	NU
					

Implementation rate of health education	-0.17	-0.18	-0.04	-0.03	-0.10	-0.08
	NU	NU	NU	NU	NU	NU

Implementation rate of health counseling	-0.13	-0.06	-0.15	-0.03	-0.15	0.07
	NU	NU	NU	NU	NU	NU

Implementation rate of home-visit guidance	-0.20*	-0.12	-0.06	-0.07	0.03	-0.12
	NS	NU	NU	NU	NU	NU

Ratio of public health nurses	-0.22*	-0.21*	-0.26^†^	-0.22*	0.03	-0.10
	NS	NS	-0.22*	-0.22*	NU	NU

Population density	0.36^‡^	0.35^‡^	0.16	0.18	0.03	0.03
	NS	0.23*	NU	NU	NU	NU

Per capita income	0.15	0.08	0.26^†^	0.05	-0.07	0.08
	NU	NU	NS	NU	NU	NU

Ratio of hospital beds	-0.07	-0.21*	0.32^†^	-0.05	0.13	-0.06
	NU	NS	NS	NU	NU	NU

Ratio of physicians	0.21*	0.06	0.32^†^	0.15	0.11	0.05
	0.24*	NU	0.29^†^	NU	NU	NU

Multiple correlation coefficient						
	0.44^‡^	0.42^‡^	0.39^‡^	0.22*	-	-

Upper shows correlation coefficient; lower, standardized regression coefficient. NU, not used for stepwise regression analysis; NS, not selected by stepwise regression analysis.

*p < 0.05, ^†^p < 0.01, ^‡^p < 0.001.

Certification rates for long-term care insurance services were 3.0% for those from 65 to 74 years old and 19.6% for those 75 years and over. The implementation rate of health counseling correlated positively with the certification rate for long-term support need, and the ratio of public health nurses correlated positively with that for long-term care need grade 1 (Table 6). The implementation rate of home-visit guidance correlated negatively with certification rates for long-term care need grades 2 and 3. The implementation rate of health counseling correlated negatively with the certification rate for long-term care need grade 4. The certification rate for long-term care need grade 4 correlated positively with medical expenses for cerebrovascular diseases (r = 0.37, p < 0.01).

**Table 6.** Relationship of health services, medical supplies and socioeconomic factors to certification rates for the long-term care insurance services.

	Support need	Care need Grade 1	Care need Grade 2	Care need Grade 3	Care need Grade 4	Care need Grade 5
Visit rate for basic health examinations	0.09	0.13	0.04	-0.10	0.00	0.12
	NU	NU	NU	NU	NU	NU

Implementation rate of health education	0.29^†^	0.38^‡^	-0.20*	-0.26^†^	-0.37^‡^	-0.08
	NS	NS	NS	NS	NS	NU

Implementation rate of health counseling	0.34^‡^	0.43^‡^	-0.15	-0.28^†^	-0.37^‡^	0.04
	0.34^‡^	NS	NU	NS	-0.37^‡^	NU

Implementation rate of home-visit guidance	0.21*	0.47^‡^	-0.25*	-0.30^†^	-0.30^†^	-0.08
	NS	NS	-0.25*	-0.30^†^	NS	NU

Ratio of public health nurses	0.31^†^	0.56^‡^	-0.15	-0.30^†^	-0.14	0.13
	NS	0.56^‡^	NU	NS	NU	NU

Population density	-0.20*	-0.20	0.15	0.16	0.18	-0.02
	NS	NU	NU	NU	NU	NU

Per capita income	-0.14	-0.00	-0.05	-0.06	-0.02	-0.19
	NU	NU	NU	NU	NU	NU

Ratio of hospital beds	-0.05	0.03	-0.04	-0.06	0.17	0.09
	NU	NU	NU	NU	NU	NU

Ratio of physicians	-0.08	-0.04	-0.10	-0.06	0.15	0.07
	NU	NU	NU	NU	NU	NU

Multiple correlation coefficient						
	0.34^‡^	0.56^‡^	0.25*	0.30^†^	0.37^‡^	-

Upper shows correlation coefficient; lower, standardized regression coefficient. NU, not used for stepwise regression analysis; NS, not selected by stepwise regression analysis.

*p < 0.05, ^†^p < 0.01, ^‡^p < 0.001.

## DISCUSSION

The author used medical expenses for the NHI as an index because the subjects of the NHI aged 40 years and older are included in the subjects in the municipalities using health services mandated by the Health and Medical Service Law for the Elderly. The exact number of the latter subjects can be identified by a survey of entire households. Therefore, the visit rates for health examinations are often inaccurate in large municipalities. The CV indicates that annual fluctuations of many indices used in this study are not significant except for the medical expenses for each malignant neoplasm. Some municipalities have not conducted lung cancer screenings because there are questions about whether it is effective^15^^)^. However, all municipalities have performed chest X-ray radiography for tuberculosis screening with a higher visit rate than cancer screening^16^^)^.

Implementation rates of the health services, including ratios of public health nurses, correlated positively with each other and negatively with medical expenses, independently from supplies of medical services, although the supplies of medical services correlated positively with medical expenses. Similar trends were observed by analyses on 49 municipalities with population of 5,000 - 29,999. Among the health services, visit rates for health examinations, implementation rates for health education or health counseling, or ratios of public health nurses were selected by stepwise regression analysis using medical expenses as the dependent variables. However, the individual effect of each health service cannot be clarified because the implementation rates of these services confound each other. A positive relationship between the ratio of public health nurses and medical expenses for hypertensive diseases suggests the results of finding patients by public health nurses and their advising to the patients to consult physicians. This study showed that health services affect medical expenses in several disease groups. The effects of health services on medical expenses for the elderly have already been reported^3^^,^^7^^)^. Indices of medical supplies correlated with medical expenses, as reported by other investigators^17^^)^. Only medical expenses for hypertensive diseases showed a stronger correlation with the ratio of physicians in the municipalities than in the secondary emergency medical service areas. The results suggest that patients with hypertension consult clinics that are close to their homes^18^^)^. Population density correlated with both medical expenses and medical supplies. Thus, it was difficult to establish the meaning of population density. Among the cancer screenings, the effect of uterine and breast cancer screenings on medical expenses is difficult to see because the visit rates are lower than the national goals in many municipalities. The negative correlation between health services and medical expenses was quite clear when medical expenses were standardized by age group. The author determined that health services reduced medical expenses by prolonging life expectancy and shortening the periods during which patients were bedridden^19^^)^.

Implementation rates of the health services correlated positively with the certification rates for minor long-term care needs and negatively with those for serious long-term care needs. The author confirmed that municipalities that emphasize health services use early intervention for patients who need long-term care services in an effort to prevent aggravation of their health conditions. The relationship between the certification rate for serious long-term care needs and medical expenses for cerebrovascular diseases suggests that cerebrovascular disease is an important cause of serious long-term care needs. The relationship of health services to the expenses for long-term care insurance, which have not been calculated yet, also should be evaluated.

Now, health promotion measures that emphasize primary prevention, such as “Healthy Japan 21,” are being conducted. Primary prevention depends on individual initiatives. However, people sometimes receive health services through a sense of civic responsibility^16^^)^. Municipalities have to support residents by providing various kinds of health services. Medical expenses are reduced by the implementation of available health services and these health services are helpful in the early detection and prevention of aggravation of disease in patients who need long-term care services. These results provide an administrative factor for the reinforcement of health services as one of the targets in the community plan of Healthy Japan 21. The author conclude, therefore, that health services must be reinforced along with primary prevention.
